# Trypsin-like serine peptidase profiles in the egg, larval, and pupal stages of *Aedes albopictus*

**DOI:** 10.1186/1756-3305-6-50

**Published:** 2013-02-27

**Authors:** Leonardo Saboia-Vahia, André Borges-Veloso, Camila Mesquita-Rodrigues, Patricia Cuervo, Geovane Dias-Lopes, Constança Britto, Ana Paula de Barros Silva, Jose B De Jesus

**Affiliations:** 1 Laboratório de Biologia Molecular e Doenças Endêmicas, Instituto Oswaldo Cruz, FIOCRUZ, Rio de Janeiro, Brazil; 2 Laboratório de Pesquisa em Leishmaniose, Instituto Oswaldo Cruz, FIOCRUZ, Rio de Janeiro, Brazil; 3 Departamento de Engenharia de Biossistemas, Universidade Federal de São João Del Rey, Minas Gerais, Brazil

**Keywords:** *Aedes albopictus*, Culicidae, Preimaginal stages, Trypsin-like serine peptidases, Peptidases, Zymography

## Abstract

**Background:**

*Aedes albopictus*, a ubiquitous mosquito, is one of the main vectors of dengue and yellow fever, representing an important threat to public health worldwide. Peptidases play key roles in processes such as digestion, oogenesis, and metamorphosis of insects. However, most of the information on the proteolytic enzymes of mosquitoes is derived from insects in the adult stages and is often directed towards the understanding of blood digestion. The aim of this study was to investigate the expression of active peptidases from the preimaginal stages of *Ae. albopictus.*

**Methods:**

*Ae. albopictus* eggs, larvae, and pupae were analyzed using zymography with susbtrate-SDS-PAGE. The pH, temperature and peptidase inhibitor sensitivity was evaluated. In addition, the proteolytic activities of larval instars were assayed using the fluorogenic substrate Z-Phe-Arg-AMC.

**Results:**

The proteolytic profile of the larval stage was composed of 8 bands ranging from 17 to 130 kDa. These enzymes displayed activity in a broad range of pH values, from 5.5 to 10.0. The enzymatic profile of the eggs was similar to that of the larvae, although the proteolytic bands of the eggs showed lower intensities. The pupal stage showed a complex proteolytic pattern, with at least 6 bands with apparent molecular masses ranging from 30 to 150 kDa and optimal activity at pH 7.5. Peptidases from larval instars were active from 10°C to 60°C, with optimal activity at temperatures between 37°C and 50°C. The proteolytic profile of both the larval and pupal stages was inhibited by phenyl-methyl sulfonyl-fluoride (PMSF) and Nα-Tosyl L-lysine chloromethyl ketone hydrochloride (TLCK), indicating that the main peptidases expressed during these developmental stages are trypsin-like serine peptidases.

**Conclusion:**

The preimaginal stages of *Ae. albopictus* exhibited a complex profile of trypsin-like serine peptidase activities. A comparative analysis of the active peptidase profiles revealed differential expression of trypsin-like isoforms among the preimaginal stages, suggesting that some of these enzymes are stage specific. Additionally, a comparison of the peptidase expression between larvae from eggs collected in the natural environment and larvae obtained from the eggs of female mosquitoes maintained in colonies for a long period of time demonstrated that the proteolytic profile is invariable under such conditions.

## Background

During the last three decades, the mosquito *Aedes (Stegomyia) albopictus* has spread from Southeast Asia to Africa, the Middle East, Europe and America. This species has demonstrated a strong ecological plasticity that allows for rapid adaptation to diversified habitats, including urban environments [1-6]. Female oviposition occurs in both natural conditions and in artificial containers, and it has been reported that *Ae. albopictus* successfully compete against other container-breeding mosquitoes, including *Ae. aegypti*[5,7]. *Ae. albopictus* is a competent vector for at least 22 arboviruses and plays significant role in the transmission of all four serotypes of the dengue virus as well as in the transmission of nematodes, such as *Dirofilaria immitis* and *Dirofilaria repens*[5,8-12].

The secretion of serine peptidases responsible for the hydrolysis of nutrients from food was first reported for *Ae. aegypti* in the early 1970s [13]. Furthermore, serine peptidases have been implicated in the regulation of immunity in *Anopheles* sp. [14-16]. Within the serine peptidase family, trypsin and chymotrypsin are the most abundant digestive enzymes in the midgut of several insect species [17]. These enzymes are characterized as having a His-Asp-Ser catalytic triad and the same basic tridimensional structure, consisting of two six-stranded β-barrels that contain the active site, the substrate recognition region and the zymogen activation domain [18]. Despite the high similarity of serine peptidases among mosquito species, each enzyme has a unique set of accessory catalytic residues that are thought to be important for determining substrate specificity [19,20].

Previous studies have reported the proteolytic activity of both trypsin and chymotrypsin in the peritrophic matrix and gut of *Ae. aegypti*, *Ae. albopictus* and *Culex quinquefasciatus* larvae [21-23]. These enzymes have also been associated with the digestion of nutrients by larvae of other diptera species, such as *Dermatobia hominis, Lutzomya longipalpis*, *Musca domestica*, and *Oxysarcodexia thornax*[24-27]. In addition, trypsins secreted in the lumen of the gut have been implicated in the process of pathogen establishment in vector mosquitoes. For example, trypsins secreted by the mosquito for food digestion are involved in the activation of the pro-enzyme form of a chitinase from the *Plasmodium* species [28-32]. Moreover, arboviruses such as the La Crosse virus (LACV; Bunyaviridae), blue tongue virus (BTV; Reoviridae) and dengue virus serotype 2 (DENV-2, Flaviviridae) use vector midgut peptidases for the proteolytic processing of viral proteins to increase viral infectivity [33-35].

The current study aims to characterize and compare the serine-peptidase proteolytic profiles of the egg, four larval instars and the pupal stage of *Ae. albopictus* using substrate SDS-PAGE zymographic analysis.

## Methods

### Chemicals

All chemicals were purchased from Sigma Chemical Company (USA). Stock solutions of Nα-Tosyl-L-lysine chloromethyl ketone hydrochloride (TLCK, 100 mM) and N-*p*-Tosyl-L-phenylalanine chloromethyl ketone (TPCK, 100 mM) were prepared in methanol. Phenyl-methyl sulfonyl-fluoride (PMSF, 250 mM) was diluted in isopropanol, whereas 1,10-phenantroline (200 mM) and pepstatin A (1 mg/ml) were dissolved in ethanol. *Trans*-epoxysuccinyl L-leucylamido-(4-guanidino) butane (E-64, 10 μM) was prepared in water. The stock solution of Z-carbobenzoxy-L-phenylalanyl-L-arginine-(7-amino-4-methylcoumarin) [Z-Phe-Arg-AMC] (3 mM) was prepared in dimethylsulfoxide (DMSO).

### Insects

Pre-imaginal stages (eggs, larvae and pupae) of *Ae. albopictus* used in the present study came from two sources: (i) a closed continuous colony (Laboratório de Transmissores de Hematozoários, Instituto Oswaldo Cruz, FIOCRUZ, Rio de Janeiro) originated from insects captured in the Brazilian state of Rio de Janeiro, and (ii) F1 generation of larvae hatched from eggs collected by ovitraps in the natural environment in endemic areas of Rio de Janeiro. In the closed colony, eggs (two days old), larvae (first larval instar, L1; second larval instar, L2; third larval instar, L3; and forth larval instar, L4) and pupae (16–20 h old) were reared at 28 ± 1°C under 80 ± 10% relative humidity, with a photoperiod of 12:12 h (LD). The larvae were kept in plastic basins containing dechlorinated water and were fed with fish food (Tetramin®). The F1 generation of larvae hatched from eggs collected by ovitraps in the natural environment in endemic areas of Rio de Janeiro was used to compare with larvae obtained from the eggs of female mosquitoes maintained for a long period of time in the closed continuous colony. The ovitrap consist in a plastic vase with a wooden oviposition paddle and hay infusion as described previously [36]. The paddles with eggs from the field were placed in plastic basins to hatch and the larvae were reared up until the fourth instar for identification using the key by Consoli & Lourenço-de-Oliveira [37]. The L4 larvae were separated from the others and reared up until imaginal stages for F1 eggs collection. F_1_ larvae were reared in the same conditions as mentioned above.

### Protein extraction and quantification

The larvae and pupa of *Ae. albopictus* were washed twice in PBS buffer (pH 7.2). Then, they were mechanically disrupted in lysis buffer containing 10% glycerol, 0.6% Triton X-100, 100 mM Tris–HCl and 150 mM NaCl. Protein was extracted from the eggs by five cycles of freezing and thawing in liquid nitrogen, followed by mechanical disruption in the same lysis buffer. The extracts were then centrifuged at 14000 × *g* for 10 minutes at 4°C to remove insoluble material. Total protein was quantified using a commercial kit (Pierce Protein assay), according to the manufacturer’s instructions.

### Zymographic assays

To investigate the proteolytic profile of total extracts, 30 μg of protein were subject to electrophoresis (110 V at 4°C) on 12% SDS-PAGE gels copolymerized with 0.1% porcine gelatin. After electrophoresis, the resulting gels were washed twice for 30 minutes at 4°C in 0.1 M sodium acetate buffer (pH 3.5 or 5.5) containing 2.5% Triton X-100, or in 0.1 M Tris–HCl buffer (pH 7.5 or 10) containing 2.5% Triton X-100. Proteolytic activity was detected after incubating the gels at 37°C in reaction buffer containing 0.1 M sodium acetate (pH 3.5 or 5.5), or in 0.1 M Tris–HCl buffer (pH 7.5 or 10). Gels loaded with egg protein were incubated for 120 minutes, whereas gels loaded with larvae protein were incubated for 30, 60, 120 or 180 minutes. Pupal enzymes were incubated for 3, 6, 12, 24, 36 or 48 h. Bands of gelatin degradation were visualized by staining the gels with 0.25% Coomassie blue R-250 and subsequent destaining with 10 % acetic acid. The molecular mass of the proteases was calculated by comparison with the mobility of a commercial molecular mass standard. All results are derived from five independent experiments carried out in triplicate.

### Effect of temperature on the proteolytic activities of larvae

The thermal sensitivity of larval peptidase activities was evaluated by incubating gels at 4°C, 10°C, 37°C, 50°C, and 60°C in 0.1 M Tris–HCl buffer (pH 7.5). Prior to gel electrophoresis, the protease extracts were incubated until they reached the proper temperature for the assay. In addition, after electrophoresis, the gels were incubated in reaction buffer that was adjusted to each temperature.

### Zymographic analysis of peptidase profiles of larvae from eggs collected from the natural environment vs. larvae from eggs of a colony maintained for a long period

For this assay, two different types of *Ae. albopictus* larvae were used: (i) larvae from eggs collected in the natural environment in endemic areas of Rio de Janeiro and (ii) larvae obtained from the eggs of female mosquitoes maintained for a long period of time in a closed continuous colony. Larvae hatched from both types of eggs (i and ii) were prepared for zymography as previously described.

### Protease inhibition assays

Larvae and pupa homogenates were pre-incubated (before electrophoresis) for 30 min at 4°C with one of the following peptidase inhibitors: 10 μM E-64, 1 mM PMSF, 100 μM TLCK, 100 μM TPCK, 10 μM pepstatin-A or 10 mM 1,10-phenanthroline. After electrophoresis, each inhibitor, at the same concentration, was also added to the reaction buffer. Peptidase activities were then resolved as described previously.

### In-solution enzymatic assays

Peptidase activity from larval homogenates was determined by in-solution assays using the fluorogenic substrate Z-Phe-Arg-AMC in the presence or absence of TLCK. One hundred micromolar of substrate was used as a working solution for each assay. The reactions were initiated by diluting 10 μg of protein from the larvae of each instar in 100 mM sodium phosphate buffer (pH 7.5). The fluorescence intensity was measured every 5 min during 60 min by spectrophotofluorometry (SpectraMax Gemini XPS, Molecular Devices, CA) using excitation and emission wavelengths of 380 and 460 nm, respectively. All assays were performed at 37°C and pH 7.5. Controls lacking either the enzyme or the substrate were also included. All results are derived from three independent experiments performed in triplicate.

## Results

### Time course of proteolytic activities from larval instars and influence of pH and temperature on the zymographic profile

**Figure 1:**
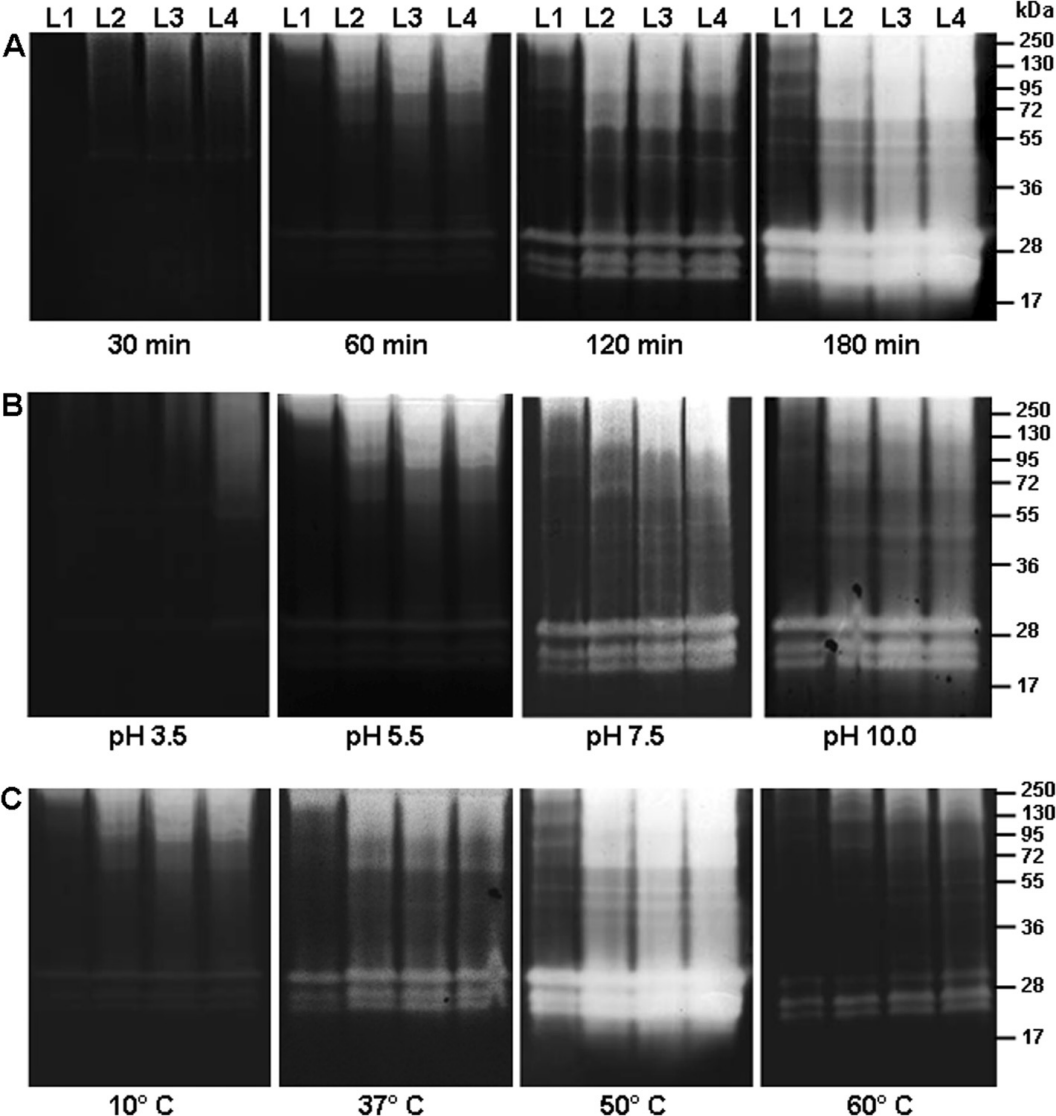
**Time course of the proteolytic activities exhibited by the four larval instars of *Ae. albopictus *and influence of pH and temperature on the proteolytic profiles. A**. Proteolytic activities were evaluated after 30 min, 60 min, 120 min and 180 min of incubation in 0.1 M Tris–HCl buffer (pH 7.5). **B**. The effect of pH on the proteolytic activities was evaluated by incubation at 37°C for two hours in 0.1 M sodium acetate buffer pH 3.5, 5,5 or 0.1 M Tris–HCl buffer pH 7.5, 10.0. **C**. Enzyme activities were detected after incubating the gels for 2 hours at 10°C, 37°C, 50°C, and 60°C in 0.1 M Tris–HCl buffer (pH 7.5). L1, first larval instar; L2, second larval stage; L3, third larval instar; and L4, fourth larval instar. The numbers on the right indicate the apparent molecular masses of the peptidases (kDa).

The zymographic profile from L1, L2, L3 and L4 larval instars was analyzed after 30, 60, 120 and 180 minutes of incubation at 37°C and pH 7.5 to evaluate the influence of time on their enzymatic activities (Figure 1A). We found that the intensity of proteolytic activities increased progressively from 30 to 180 minutes of incubation. The proteolytic profile was composed of at least eight bands ranging in size from 17 to 130 kDa. The influence of pH on the proteolytic activities of larval instars was evaluated after incubation of the gels for two hours in different reaction buffers ranging from pH 3.5 to 10 (Figure 1B). Although enzymatic activities were detected at all pH condition, the intensity of peptidase activities was reduced at pH 3.5 and 5.5. The thermal sensitivity of peptidases from larval homogenates was measured at pH 7.5 and temperatures of 4°C, 10°C, 37°C, 50°C and 60°C. At 4°C little activity was observed (data not shown) whereas at 10°C the proteolytic activities were less intense compared to those visualized at the standard temperature 37°C. At 50°C, the enzymatic activities were strongly increased to the extent that some of the proteolytic halos overlapped in the L2, L3 and L4 instars. Conversely, some bands between 40 kDa and 130 kDa were more visible at 50°C in the L1 instar. At 60°C, the enzymatic activities were reduced compared to the standard condition (Figure 1C).

### Peptidase inhibition assays in larval instars and in-solution detection of proteolytic activity

**Figure 2:**
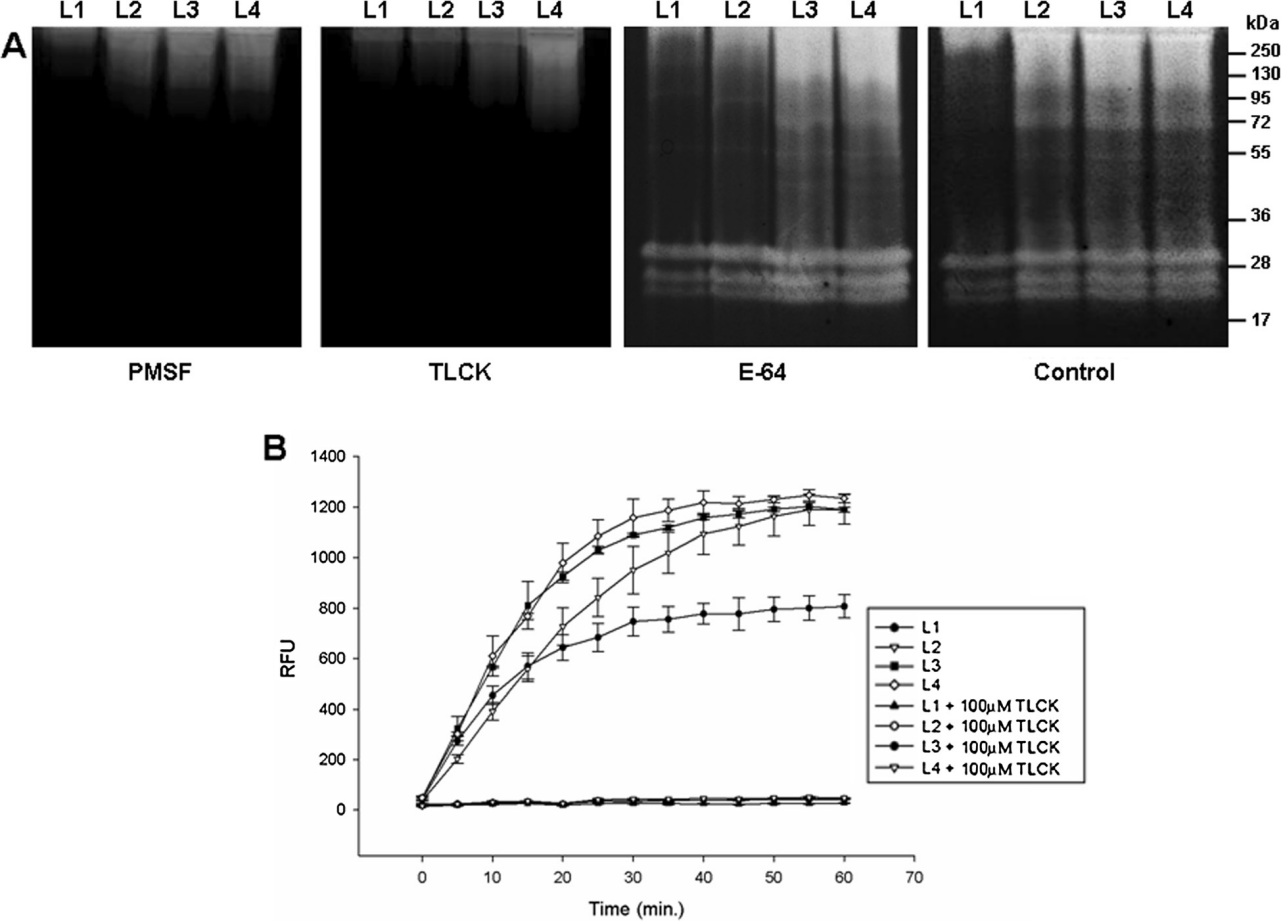
**Effect of peptidase inhibitors on the proteolytic profiles of the four larval instars of *Ae. albopictus*, and quantitative in-solution assays of the proteolytic activities. A**. Samples were pre-incubated for 30 min in the presence of 1 mM PMSF, 100 μM TLCK and 10 μM E-64. The proteolytic activities were detected after incubating the gels for 2 hours at 37°C in Tris–HCl buffer (pH 7.5). The control was processed under the same conditions but in the absence of inhibitors. **B**. The in-solution assays were performed using the fluorogenic substrate Z-Phe-Arg-AMC in the absence (control) or presence of 100 μM TLCK in 100 mM sodium phosphate (pH 7.5). L1, first larval instar; L2, second larval stage; L3, third larval instar; and L4, fourth larval instar. The numbers on the right indicate the apparent molecular masses of the peptidases (kDa).

The proteolytic activities of all larval instars were inhibited by 1 mM PMSF and 100 μM TLCK (Figure 2A). The zymographic profile was not affected by 10 μM E-64 (Figure 2A), 100 μM TPCK, 10 μM pepstatin A or 10 mM 1,10-phenanthroline (data not shown). Protein extracts from larval instars were used to determine in-solution peptidase activities using the fluorogenic substrate Z-Phe-Arg-AMC in the presence or absence of TLCK, a specific inhibitor of trypsin-like serine peptidases. The enzymatic activities increased progressively from L1 to L4, and each instar displayed different velocities of substrate degradation. All enzymatic activities were strongly inhibited by TLCK (Figure 2B).

### Comparative analysis of the peptidase profiles of larvae from eggs collected from the natural environment vs. larvae from eggs collected from a colony maintained for a long period of time

**Figure 3:**
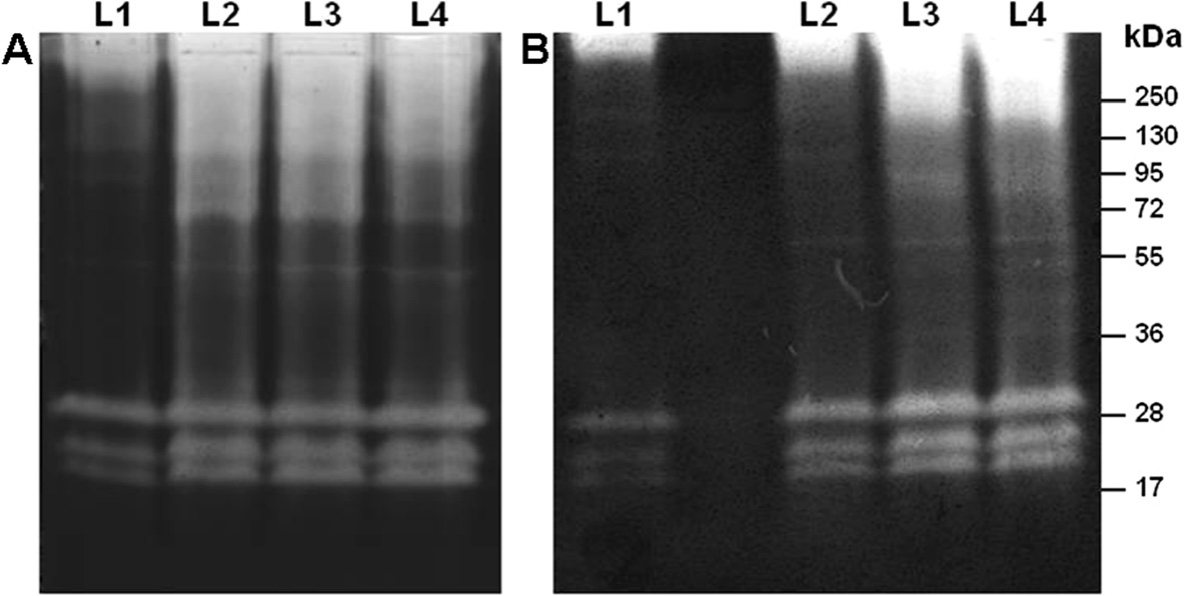
**Comparative analysis of the proteolytic profile of *Ae. albopictus *larvae from (A) eggs obtained from the natural environment in endemic regions of Rio de Janeiro and (B) eggs obtained from colonies maintained for a long time period.** Proteolytic activities were detected after incubating the gels for 2 hours at 37°C in 0.1 M Tris–HCl buffer (pH 7.5). The numbers on the right indicate the apparent molecular masses of the peptidases (kDa).

To evaluate the stability of the peptidase activities detected by zymography, the proteolytic profile from larval instars was verified by comparing the enzymatic activities of larvae from eggs collected in the natural environment and larvae obtained from eggs collected from female mosquitoes maintained in colonies for a long period of time. The larval instars obtained from eggs under both conditions exhibited similar proteolytic profiles (Figure 3).

### Comparison of peptidase profiles from eggs and larval instars

**Figure 4:**
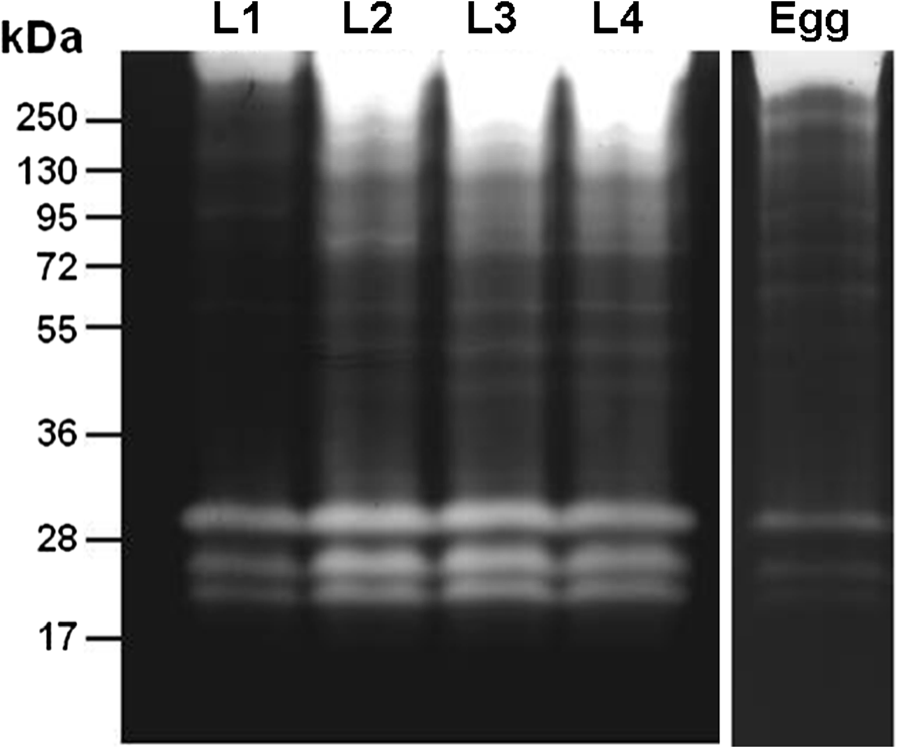
**Comparison between the proteolytic profiles of eggs and larvae of *Ae. albopictus*.** Proteolytic activities were detected by incubating the gels for 2 hours at 37°C in 0.1 M Tris–HCl buffer (pH 7.5). The numbers on the left indicate the approximate molecular masses of the peptidases (kDa).

The proteolytic profile displayed by eggs exhibited bands between 17 and 130 kDa. The intensity of the proteolytic activities from eggs was lower than those from larvae; however, bands ranging from 72 to 130 kDa were better visualized in the egg extracts compared to the larval extracts. The high molecular weight bands were not well resolved in the proteolytic profiles of the larval instars, likely due to the extremely high enzymatic activity in this area (Figure 4).

### Time course of proteolytic activities from the pupal stage and influence of pH on the zymographic profile

**Figure 5:**
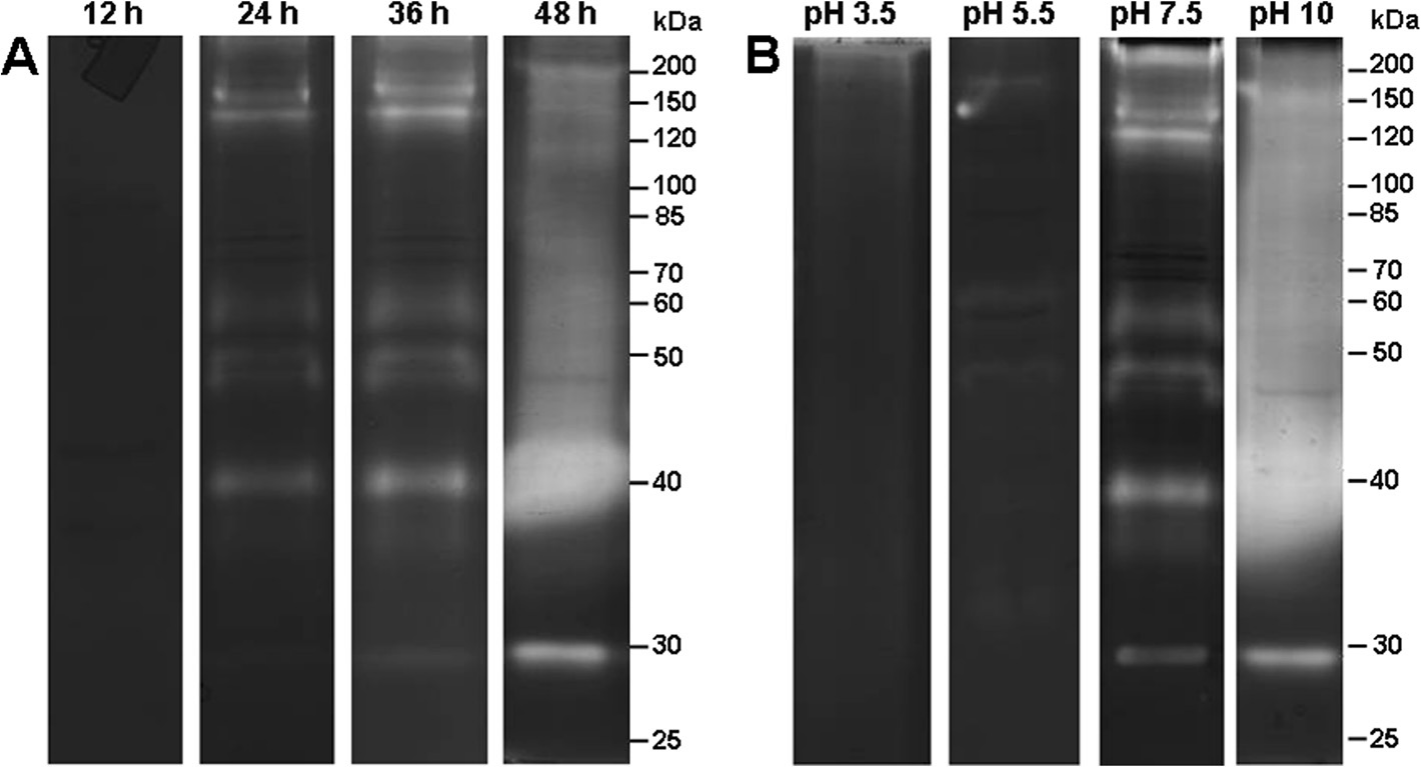
**Time course of the proteolytic activities of *Ae. albopictus *pupae and influence of pH on the proteolytic profile. A**. Peptidase activities were detected after 12, 24, 36 and 48 h incubation of the gels at 37°C in 0.1 M Tris–HCl buffer (pH 7.5). **B**. The effect of pH on the proteolytic activities was evaluated by incubation at 37°C for 36 hours in 0.1 M sodium acetate buffer pH 3.5, 5,5 or 0.1 M Tris–HCl buffer pH 7.5, 10.0. The numbers on the right indicate the apparent molecular masses of the peptidases (kDa).

Proteolytic activity from the pupal stage was assessed after 3, 6, 12, 24, 36 and 48 h of incubation at 37°C and pH 7.5. Enzymatic activity was not detected after 3, 6 or 12 h of incubation (data not shown and Figure 5A). In contrast, the intensity of the proteolytic activity increased progressively from 24 to 48 h (Figure 5A). The peptidase profile was well-resolved after 36 h of reaction, with nearly six evident bands of activity detected between 30 and 200 kDa. The proteolytic profile from the pupal stage was analyzed after 36 h of incubation in different buffers at pH 3.5, 5.5, 7.5 and 10 (Figure 5B). No enzymatic activity was observed at pH 3.5, while weak proteolytic activity was detected at pH 5.5. The peptidase profile was best resolved at pH 7.5. At pH 10, the proteolytic halos of gelatin degradation overlapped, making it impossible to visualize defined bands, except for one band migrating at 30 kDa.

### Peptidase inhibition assays in the pupal stage

**Figure 6:**
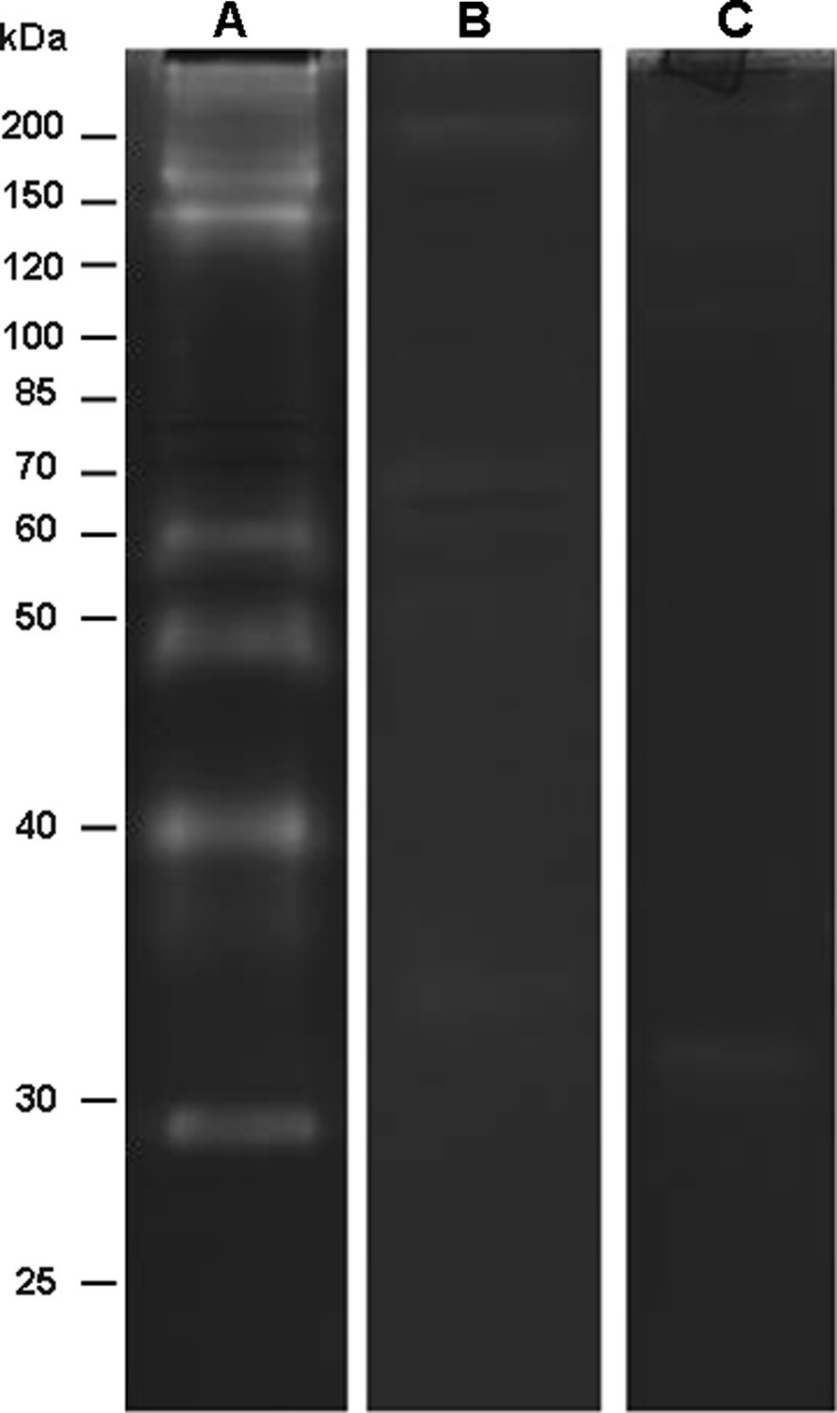
**Effect of TLCK and PMSF on the profile of the proteolytic extracts from *Ae. albopictus *pupae.** Proteolytic activities were detected after incubating the gels for 36 hours at 37°C in Tris–HCl buffer (pH 7.5). Enzymatic assays were performed in the absence of peptidase inhibitors (**A**), in the presence of 100 μM TLCK (**B**), and in the presence of 1 mM PMSF (**C**). The numbers on the left indicate the approximate molecular masses of the peptidases (kDa).

The enzymatic profile exhibited by pupal homogenates was strongly inhibited by 1 mM PMSF and 100 μM TLCK (Figure 6). Proteolytic activities were not affected by 10 μM E-64, 100 μM TPCK, 10 μM pepstatin A, or 10 mM 1,10-phenantroline (data not shown).

## Discussion

Most of the information on the proteolytic enzymes of mosquitoes is comprised from data collected at the adult stages and is often directed towards the understanding of blood digestion [38-40]. The aim of this study was to investigate the expression of active peptidases from the preimaginal stages of *Ae. albopictus*. To this end, we characterized the proteolytic profile from whole extracts of eggs, larvae and pupae using SDS-substrate gel. The peptidase activities were dependent on the time, pH and temperature of incubation. The proteolytic profile from all of the analyzed stages was composed primarily of trypsin-like serine peptidases, and each stage exhibited a specific and complex profile of these enzymes. Additionally, a comparison of the peptidase expression between the larvae from eggs collected from a natural environment and larvae obtained from eggs of female mosquitoes maintained in colonies for a long period of time showed that the proteolytic profile is invariable under these conditions.

Although the genome of *Ae. albopictus* has not yet been completely sequenced, the expression of multiples genes coding for trypsin-like serine peptidases has been reported in the genome of different mosquitoes species, such as *Anopheles gambiae*, *Ae. aegypti* and *Culex quinquefasciatus*, accounting for 345, 380 and 403 putative genes, respectively [41-44]. Compared to other diptera species, such as *Drosophila melanogaster*, which has 260 genes encoding trypsin-like serine peptidases, the mosquito genome exhibits a larger genetic repertoire of these genes, likely due to gene expansion events [45,46]. The maintenance of a variety of trypsin-like coding genes may be related to their key role in numerous physiological processes of the insect, such as digestion, immunity, reproduction, development, signal transduction and wound healing [47-52]. However, the characterization of active trypsin-like peptidases is scarce, and little is known about the biochemical nature of these enzymes in the preimaginal stages.

In the present study, we observed that *Ae. albopictus* larval instars displayed a clear proteolytic pattern that was detected after 2 hours of reaction. In contrast, it has been reported that *Ae. aegypti* exhibits a zymographic profile after only 1 hour of reaction [22]. These results suggest that the larval peptidases in these species present (i) distinct kinetic activities or (ii) differential quantitative expression. Such differences could also be related to species-specific nutritional characteristics of the larvae. In this regard, the larvae of *Ae. albopictus* feed more slowly and eat smaller amounts of nutrients at one time compared to the larvae of *Ae. aegypti*[21]. This slower intake of food could lead to slower or reduced activation/expression of peptidases involved in nutrient digestion. Post-feeding induction of peptidase expression has been observed, particularly in adults of *Ae. aegypti*[53-55]. Here, zymographic analyses revealed that the expression of peptidases increases at each larval instar. This result was corroborated by the quantitative in-solution assays using the fluorogenic substrate Z-Phe-Arg-AMC. Quantitative differential expression in larval instars has also been observed by other authors, specifically in the intestine, using alternative methods [21,39,56]. In the present study, the proteolytic classes of the enzymes were characterized using different inhibitors, revealing that peptidases from the four larval instars were inhibited by PMSF, an inhibitor of trypsin and chymotrypsin, and TLCK, an inhibitor of trypsin. These results indicate that the enzymes were predominantly trypsin-like serine peptidases in larval instars of *Ae. albopictus*, which is in agreement with previous descriptions that suggest the occurrence of trypsin in other species from diptera [13,21,24,25,57]. Although no other peptidase classes were detected under the conditions used here, we cannot rule out the possibility that other classes of peptidases could be detected under different experimental conditions.

The four larval instars of *Ae. albopictus* showed proteolytic activity in a broad range of pH values, with optimal activity between pH 7.5 and 10. Accordingly, the larvae of *Ae. aegypti*, *An. stephensi* and *Cx. quinquefasciatus* displayed high enzymatic activities at an alkaline pH [22,23,58]. These strong proteolytic activities may be associated with larval nutrition because these stages are highly detritivorous and need to eat large amounts of food to obtain their basic nutrients. Indeed, the main peptidases expressed by larvae are thought to be involved in the processing of nutrients and have optimal activity at an alkaline pH [13,58]. In addition, we demonstrated that larval instars exhibit a complex profile of active peptidases composed by at least eight bands of trypsin-like serine peptidases. These results are in agreement with a recent transcriptome study showing that 12 serine peptidases-like genes were preferential expressed in the larvae of *Ae. aegypti*[39,59]. The broad spectrum of enzymatic activities detected in the larvae of *Ae. albopictus* could be related to their survival in aquatic environments that are lacking nutrients [13,58].

In addition, the zymographic patterns of larvae from *Ae. albopictus* and *Ae. aegypti*[22] share a band of proteolytic activity with the same intensity at approximately 28 kDa. Additionally, several bands between 36 and 72 kDa observed in the enzymatic profile of *Ae. albopictus* match those of *Ae. aegypti*, although with different intensities. These data could indicate that (i) the expression of genes coding for some active trypsin-like serine peptidases isoforms is conserved among *Aedes* species and (ii) that other isoforms are species-specific. In fact, this conservation is expected given the importance of these enzymes for the life cycle of these mosquitoes [21,56,59]. On the other hand, bands of proteolytic activities with different intensities within the same stage of development, among distinct stages and between the two species may indicate that some genes coding for trypsin are differentially expressed or that some isoforms with specific catalytic features are differentially regulated during the life cycle of the insect. Complex mechanisms regulating the expression of trypsin in insects have been previously described [39,53-56,60].

We next sought to investigate the stability of the proteolytic profile in larvae obtained from the eggs of females reared in a colony for long time periods compared with larvae hatched from eggs collected in the natural environment. Our results demonstrate that the enzymatic pattern did not change, suggesting that the qualitative expression of peptidases is stable under the experimental conditions used here. In other words, such stability suggests that the genes coding for these enzymes could be under strong selective pressure so that the proteolytic profile is maintained in both natural and colony conditions. These results indicate that inbreeding during prolonged maintenance of the colony does not alter the expression of the genes coding for the trypsin-like serine peptidases detected in zymographic assays of the larvae. In addition, such observations suggest that trypsin-like serine peptidases of *Ae. albopictus* could be constitutively expressed during all developmental stages.

The proteolytic profile of *Ae. albopictus* eggs was similar to that exhibited by the larval instars, specifically the L1 instar, in the number and intensity of bands. Three bands of enzymatic activity between 17 and 28 kDa were observed in both the eggs and larvae, although they displayed different intensities. The similarity of the active peptidase profile observed between the eggs and L1 may be explained by the fact that the eggs were assayed at the final stage of maturation when the embryo is very similar to the young L1. In addition, the proteolytic activities from the eggs likely had lower intensity because before they hatched, the larvae were lethargic, presenting low metabolism inside the eggs.

The pupal stage exhibited a complex and stage-specific proteolytic profile, composed of six bands of activity. Compared to the larval profile, which showed activities in a wide pH range (3.5 – 10), the pupal stage presented activities exclusively between pH 7.5 and 10. Peptidases from the larval extracts could be observed after 2 hours of incubation, whereas peptidases from the pupal stage were detected only after 24 hours of reaction. These observations indicate a reduction in the expression of active digestive peptidases after the last larval molt [13]. However, although pupae do not feed, serine peptidase activities could be related to the proteolysis of the remaining larval tissue during metamorphosis. In support of this idea, in *Sarcophaga peregrina*, a 26 kDa trypsin protein was isolated from the corpus luteum, an organ that develops temporarily in the pupae and serves to disintegrate the gut of immature stages and reshape it to form the adult insect's gut [50,61-63].

The effect of temperature on trypsin-like serine peptidase activities from larvae was also tested. When compared to the control condition (37°C), the proteolytic activities of all larval instars were susceptible to low temperatures (4°C and 10°C) and highly stimulated at high temperatures (50°C). Although enzymatic activity decayed at 60°C, a clear profile could still be observed at this temperature. The strong decrease of proteolytic activity could be due to the thermal denaturation of the enzymes. Similar results were described for trypsin and chymotrypsin from the larvae of *Tenebrio molitor*[64,65] and for serine peptidases from *Oestrus ovis*[66] and *Ae. aegypti*[22]. Because temperature is one of the most important environmental conditions for sustaining life on earth, the adaptation of an organism to extreme environments requires the optimization of its enzymatic repertoire. In this sense, the investigation of thermostable proteins, which are highly conserved in phylogenetically related groups of organisms, could help to identify changes in amino acid sequences that could be associated with thermal adaptation [67]. Because *Ae. aegypti* and *Ae. albopictus* are related species that originated from different environments, a detailed study comparing the sequence and structure of the trypsin isoforms could provide important information on the molecular basis of the thermal stability of these enzymes.

## Conclusion

The zymographic profiles of the preimaginal stages of *Ae. albopictus* are composed of a complex combination of trypsin-like serine peptidase activities that exhibit stage-specific characteristics. A comparison of the proteolytic activities during different developmental stages allowed for the detection of both qualitative and quantitative differences in trypsin activities, indicating that the serine peptidases are expressed in a stage-specific manner. In addition, we demonstrate that the proteolytic profile in larval instars is stable because larvae from the natural environment and larvae from a colony exhibited identical trypsin-like peptidase patterns. Such a phenotypic characteristic could be exploited for the characterization of other Culicidae insects.

## Competing interests

The authors declare that they have no competing interests.

## Authors’ contributions

JBJ, LSV and PC designed the study. LSV, ABV, CMR, APB and GDL performed the experimental work. LSV, ABV, CMR, PC and JBJ analyzed the data and prepared the manuscript, with critical input from CB. All of the authors read and approved the final manuscript.
